# Practical strategies for the evaluation of high-affinity protein/nucleic acid interactions

**DOI:** 10.4081/jnai.2013.e3

**Published:** 2013-09-20

**Authors:** Sarah E. Altschuler, Karen A. Lewis, Deborah S. Wuttke

**Affiliations:** Department of Chemistry and Biochemistry, University of Colorado, Boulder, CO, USA

**Keywords:** high-affinity, protein, nucleic acid, EMSA, filter binding

## Abstract

The quantitative evaluation of binding interactions between proteins and nucleic acids is highly sensitive to a variety of experimental conditions. Optimization of these conditions is critical for obtaining high quality, reproducible data, particularly in the context of very high affinity interactions. Here, we discuss the practical considerations involved in optimizing the apparent binding constant of an interaction as measured by two common quantitative assays, electrophoretic mobility shift assay and double-filter binding when measuring extremely tight protein/nucleic acid interactions with sub-nanomolar binding affinities. We include specific examples from two telomere end-binding protein systems, *Schizo -saccharomyces pombe* Pot1 and *Saccharomyces cerevisiae* Cdc13, to demonstrate potential experimental pitfalls and some useful strategies for optimization.

## Introduction

A variety of robust methods are available to quantitatively evaluate the affinity of one macromolecule for another. These approaches are either equilibrium or non-equilibrium techniques, depending on how the bound complex is distinguished from the unbound components. In many of these strategies, the target and titrant molecules are first mixed to establish a binding equilibrium, which is determined by the rates of complex formation (*k*_on_) and complex dissociation (*k*_off_). Ideally, all binding interactions would be measured under equilibrium conditions, using methods such as sedimentation equilibrium, equilibrium dialysis, fluorescence polarization, or isothermal titration calorimetry (ITC).^1–4^ However, these approaches are frequently reagent limited, often requiring micromolar to millimolar concentrations of reagents. Furthermore, for very high-affinity interactions (sub-nanomolar *K*_D,app_ values), the binding can be technically impossible to measure due to sensitivity issues. For example, the amount of heat evolved by tight binding interactions at the concentrations required to make the measurement can be too small to measure by ITC.^4^

High-affinity protein/nucleic acid interactions lend themselves well to easily accessible non-equilibrium techniques such as electrophoretic mobility shift assay (EMSA) and double-filter binding.^5–7^ These assays require much smaller quantities of reagents than many equilibrium techniques and the sensitivity achieved with ^32^P-radiolabeling of the nucleic acid target allows for the measurement of low picomolar *K*_D,app_ values. While EMSA and double-filter binding are powerful tools, they are also highly susceptible to experimental artifacts, particularly in the regime of very tight binders in which small differences in *K*_D,app_ values can produce relatively large standard errors. Here, we will discuss strategies to optimize experimental conditions for these two assays in order to measure accurate *K*_D,app_s and reduce uncertainty in the measurements in this regime. To demonstrate these practical methods for experimental execution, we discuss two different systems that exemplify the simplest target/titrant system: one-to-one stoichiometry with no cooperativity. More complex systems have been addressed elsewhere, including cooperativity, which has been assessed by both EMSA and filter binding techniques.^8–11^ In our laboratory, we have extensively studied the binding activity of two single-stranded DNA (ssDNA) telomere end-binding protein systems, *Schizosaccharomyces pombe* Pot1 (*Sp*Pot1) and *Saccharomyces cerevisiae* Cdc13.^12–14^ These proteins tenaciously bind their respective cognate ssDNA ligands with apparent dissociation constants in the low picomolar range (K.A.L and D.S.W., unpublished results) (Table 1).^8,14,15^ In the case of *Sp*Pot1, the dual OB-fold DNA-binding domain can additionally be separated into two subdomains that independently bind telomeric ssDNA and can be compared to the full-length protein.^8,11,13,16–18^ While the evaluations of these extraordinarily tight binding regimes necessitate some particular accommodations, all of the basic conditions and parameters discussed below are also applicable to weaker interactions between proteins and nucleic acids.

The experimental determination of binding *K*_D_s employs the binding isotherm.^19,20^ Determining the exact concentrations of free and bound titrant and target molecules in solution is not always possible; however, it is usually experimentally feasible to determine the fraction of total titrant bound to target ([DP]/[D]_T_). Therefore, a derivation of the binding equation is commonly used that relates the fraction of target bound to a term comprised of the concentration of free titrant and the *K*_D_, hereafter referred to as Eq. 1:

(1) $$\frac{\left[\text{DP}\right]}{{\left[D\right]}_{T}}=S\left(\frac{\left[P\right]}{[P]+K_{D,\text{app}}}\right)+O$$

where [D]_T_ is the total concentration of target (nucleic acid), [P] is the free titrant concentration (protein), [DP]/[D]_T_ is the fraction of nucleic acid bound, S is a saturation offset, *K*_D,app_ is the calculated apparent dissociation constant, and O is a background offset. Importantly, experimental data produce strictly *apparent* dissociation constants. This formulation of the binding isotherm is only applicable to simple two-state binding systems, to which we are limiting the discussion below. To further simplify the implementation of this theory, the experimental conditions are set such that [P]_Free_ ≈ [P]_T_ to obviate the need to directly measure [P]_Free_. This is accomplished by holding constant the total concentration of target ([D]_T_) well below the *K*_D,app_. The protein is titrated over a large concentration range spanning concentrations well above and below the *K*_D,app_. The fraction bound is quantitated and plotted against protein concentration, and then fitted to Equation 1 to solve for *K*_D,app._ There exist extensive resources for least-squares curve fitting for both simple and complex systems, as well as methods for evaluating the quality of those fits.^9,21,22^ When working with an extremely tight protein/nucleic acid system, the application of this strategy to experimental design presents a variety of challenges. Here, we describe practical strategies to optimize such systems to promote protein stability and activity and to produce replicable data that have reasonable signal-to-noise ratios for accurate quantitation and high confidence intervals. Small *K*_D,app_ values require low protein concentrations and even lower nucleic acid concentrations, both of which present signal-to-noise concerns. We will first discuss concerns that are specific to either the EMSA or double-filter binding technique, and then address considerations that are generally applicable to both of these assays as well as data analysis when working with a very high-affinity system.

## Materials and Methods

All assays discussed used standard de-salted ssDNA oligonucleotides (Integrated DNA Technologies). We have found that for DNA oligonucleotides between 6 and 30 nt, standard de-salted oligonucleotides are typically of equal purity to those that have been PAGE- or HPLC-purified (*data not shown*), a consideration that is helpful when studying the binding specificities of protein/nucleic acid systems in which dozens of different sequences may be required. ssDNA oligonucleotides were 5′ end-labeled with ATP[γ-^32^P], 6000 Ci/mmol, 150 mCi/mL (Perkin-Elmer). The ATP[γ-^32^P] was delivered the day after the lot was synthesized, so that the actual specific activity was 8900 Ci/mmol. The oligonucleotides were labeled using T4 polynucleotide kinase (New England Biolabs), followed by purification from free ATP using MicroSpin G-25 columns (GE Healthcare). TipOne Low Retention Pipet Tips and Low Retention polypropylene microcentrifuge tubes were used for all steps of all experiments (USA Scientific). The BioRad Mini-PROTEAN system is easily adaptable for large-scale EMSA binding studies, and all EMSAs discussed here used this system. 1xTBE was prepared from a commercially available 10X stock solution (National Diagnostics); 5% glycerol was added where indicated in figure legends. Double-filter binding was conducted using a 96-well minifold vacuum manifold dot blot apparatus (GE Healthcare) and nitrocellulose and HyBond XL filters (charged nylon; GE Healthcare) were cushioned against the filter plate using a piece of 3 mm chromatography filter paper (GE Healthcare). Phosphorimaging screens (GE Healthcare) were scanned on a TyphoonXL phosphorimager (GE Healthcare). Data were quantitated using ImageQuantTL (GE Healthcare) and processed in Microsoft Excel. Kaleidagraph (Synergy Software) was used for plotting and least-squares non-linear curve fitting. Full-length *Sp*Pot1 and *Sp*Pot1-DBD were purified as described;^8^ Pot1pC was also purified as described.^12^ Full-length Cdc13 was purified essentially as described,^15^ with modifications (K.A.L., D.S.W., *et al*., in preparation).

Simulated binding data were determined as follows. Equation 1 was rearranged to yield Equation 2, which employs only fixed starting values for protein and DNA concentrations. Equation 2 was then used to calculate the fraction bound, [DP]/[D]_T_, for 15 protein concentrations for each of 12 different DNA concentrations. These 12 simulated data sets were plotted as [DP]/[D]_T_ vs. [P]_T_. Each data set was then subjected to least-squares fitting using Equation 1 to derive a fitted value for *K*_D,app_ for each DNA concentration.

## Results

### Electrophoretic mobility shift assays

**Figure 1:**
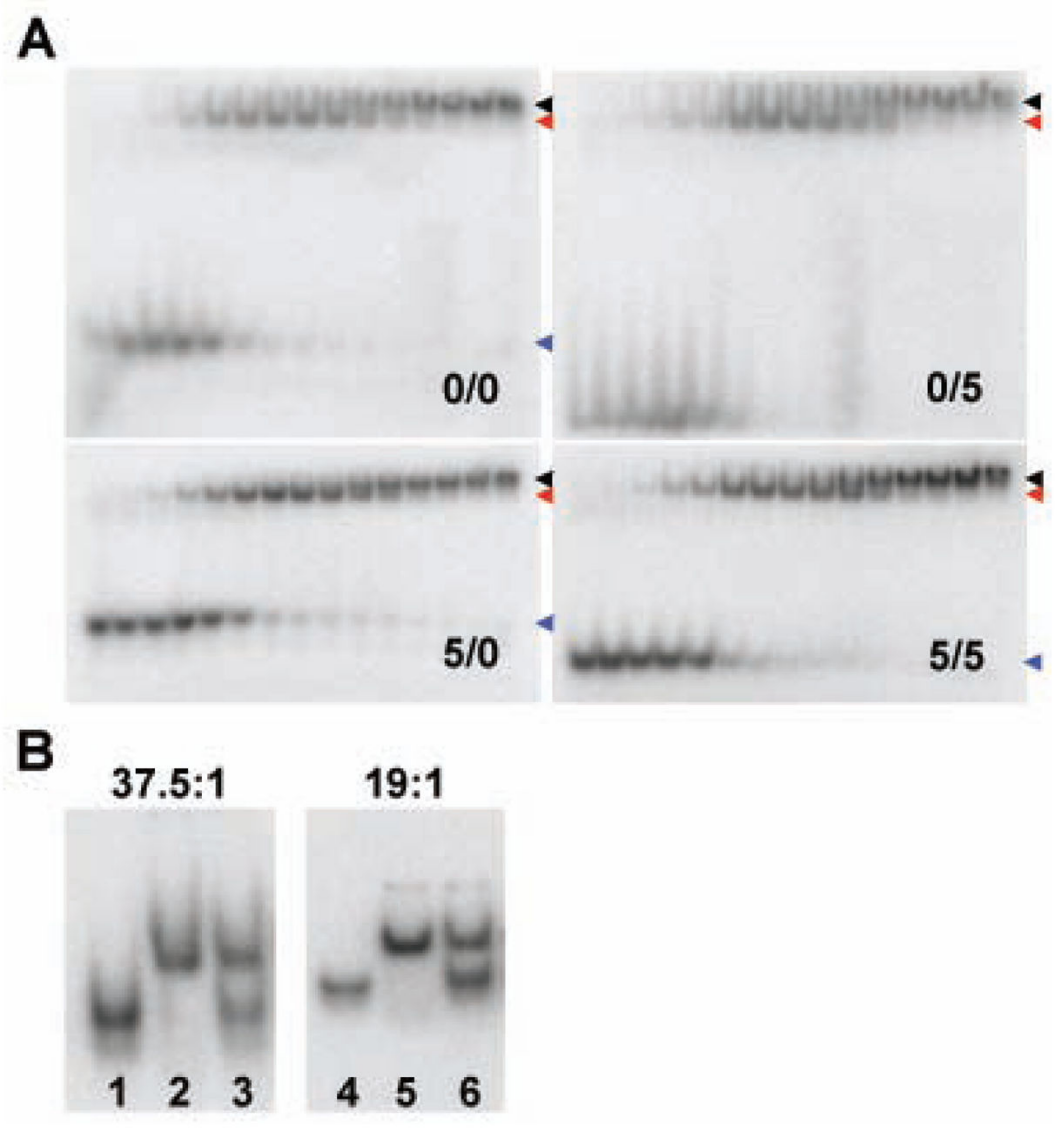
Electrophoretic mobility of species in a native gel can be affected by glycerol and the acrylamide/bisacrylamide ratio. A) S. cerevisiae Cdc13 was incubated with 1 pM Tel11 oligonucleotide and binding was conducted in 50 mM Tris, pH 7.5, 75 mM KCl, 1 mM DTT, 1 mg/mL BSA, and 15% glycerol. Cdc13 was titrated at 0.1, 0.4, 1, 4, 10, 40, 100, and 400 pM, 1, 4, 10, 40, 100, 400 nM, and 1 μM. The binding reactions were separated in 6.7% 1×TBE gels containing 0% (upper panels) or 5% (lower panels) glycerol in 1×TBE running buffer containing either 0% (left panels) or 5% glycerol (right panels), as indicated. All gels were run at 200 V for 35 minutes at 4°C. Red and black arrows indicate the monomer and dimer species, respectively, and blue arrows indicate free ssDNA. B) A lower acrylamide/bisacrylamide ratio increases band resolution. *S. pombe* Pot1-DBD (50 nM, lanes 1 and 4) and *Sp*Pot1 (50 nM, lanes 2 and 5) were incubated individually or as a mixture (lanes 3 and 6) with 50 pM 15mer ssDNA. Reactions in the left panel were run on a 10% gel made with 37.5:1 acrylamide/bisacrylamide, and those on the right panel were run on a 10% gel made with 19:1 acrylamide/bisacrylamide. The binding reactions were conducted in 20 mM Tris, pH 8.4, 50 mM NaCl, 1 mM DTT, 1 mg/mL BSA, and 15% glycerol, and both gels contained 5% glycerol and were run in 1×TBE at 200 V for 90 minutes at 4°C.

EMSA relies on the separation of unbound nucleic acid from protein-bound nucleic acid through a polyacrylamide or agarose matrix.^5,23^ The fraction of nucleic acid bound is calculated by dividing the counts from the lower mobility protein-bound species by the sum of the bound counts and those from the higher mobility free nucleic acid species (Figure 1A). Additionally, because of the rapid separation of unbound from bound species, EMSAs can identify the presence of fast off rates.^5,23^ The assay conditions should be optimized so that one can be confident in the reliability and accuracy of the data obtained. The general method and many experimental parameters have previously been expertly reviewed, and these resources should be used to design EMSA experiments.^23–25^ Here, we describe several parameters that we have identified as important considerations for large-scale EMSA studies of tight protein/ssDNA interactions, including the gel recipe, gel running conditions, and sample buffer composition.

Non-denaturing polyacrylamide gels are ideal for protein/nucleic acid separation. These gels have a base composition of 1×TBE (89 mM Tris, 89 mM boric acid, 2 mM Na_2_EDTA) and are run in 1×TBE buffer.^26,27^ This standard composition is appropriate for most protein/nucleic acid systems; however, the net charge of the protein may require the adoption of non-standard EMSA conditions, such as pH alteration or the use of agarose gels.^24,28^ Purchased pre-cast gels are often used for convenience; however, for large-scale EMSA studies, this can quickly become prohibitively expensive. The use of a multi-caster gel pouring apparatus facilitates rapid preparation of homemade mini gels, which are adaptable to the requirements of the protein/nucleic acid system under study. The optimal polyacrylamide gel recipe used is likely to be different for different systems and should be optimized prior to engaging in high-throughput studies.

A particularly important consideration in determining the gel recipe is the sizes of species to be separated. For the *Sp*Pot1 and Cdc13 telomere end-binding systems, oligonucleotide lengths typically range from 6 to 30 nucleotides and the proteins vary from ~20 kDa to >100 kDa (Table1). The net charges of these proteins provide for rapid separation of free oligonucleotides from bound complexes using a wide variety of acrylamide concentrations, which allows the gel composition to be optimized for time considerations. Lower percentage gels (<10% acrylamide) have the advantage of shorter run and gel drying times, which are important considerations for large-scale binding studies. For example, the 15mer-bound *Sp*Pot1 complex (~69 kDa) is easily resolved from free ssDNA on a 6.7% 19:1 acrylamide/bisacrylamide gel run at 200 V in under 20 min.^8^ Cdc13 dimerizes in a concentration-dependent manner, and both the monomeric and dimeric forms of Cdc13 are able to bind to the single recognition site on the oligonucleotide Tel11 (*i.e.*, only one of the protein molecules in the dimer is directly bound to Tel11). The Tel11-bound monomer and dimer species can be distinguished in the same gel conditions as the *Sp*Pot1 system in 35 minutes (Figure 1A). However, not all protein/nucleic acid complexes are so easily distinguished under standard 1×TBE gel conditions and may require optimization of pH and other variables.^24^ Additionally, some interactions are stabilized by the *caging effect*, in which the gel matrix restricts the diffusion of the dissociated components, resulting in a tighter *K*_D,app._^26^ This phenomenon is most applicable to weaker interactions, and we have not observed an effect on *K*_D,app_ with alterations of either acrylamide concentration (up to 15%) or acrylamide/bisacrylamide ratio (19:1 compared to 37.5:1). However, even with tight binding systems, these variables should be examined, and only *K*_D,app_ values obtained using the same gel recipe should be directly compared.

Optimization of band resolution is necessary to facilitate data quantitation and increase signal-to-noise. While the acrylamide/bisacrylamide ratio may not affect the *K*_D,app_, by nature of the decreased pore size, a lower ratio can increase band sharpness (Figure 1B). Band resolution can also be improved by adding 5% glycerol to the polyacrylamide gel recipe, which sharpens and intensifies the bands of both the unbound and bound species, concentrating the signal (Figure 1A). The inclusion of glycerol in the gels additionally prevents higher percentage acrylamide gels from cracking during the drying process. We have found that 5% glycerol in the electrophoresis running buffer alters how bands travel in the gel (Figure 1A), and, therefore, glycerol percentage is one of the variables to optimize to improve the quality of the gel for quantitation. Glycerol is also a critical component of the sample buffer that is used in the binding reactions, as it makes the samples dense enough to load directly onto the gel without requiring additional manipulation after the binding reaction is complete. While the glycerol concentration can be optimized for ease of loading and band clarity, the *K*_D,app_ can be affected by glycerol. For example, we have observed that the inclusion of up to 15% glycerol in the sample buffer results in the same binding constant for *Sp*Pot1/ssDNA interactions, but increasing to 17.5% inflates the measured *K*_D,app_ (1.6 pM, 1.5 pM, and 4.2 pM for 10%, 15%, and 17.5% glycerol, respectively, for the Pot1-DBD/15mer interaction; data are from a single experiment). For band resolution, species separation, and absolute affinities, the glycerol content of the gel and the electrophoresis and sample buffers is an important consideration when optimizing an assay for a specific system.

### Double-filter binding assays

**Figure 2:**
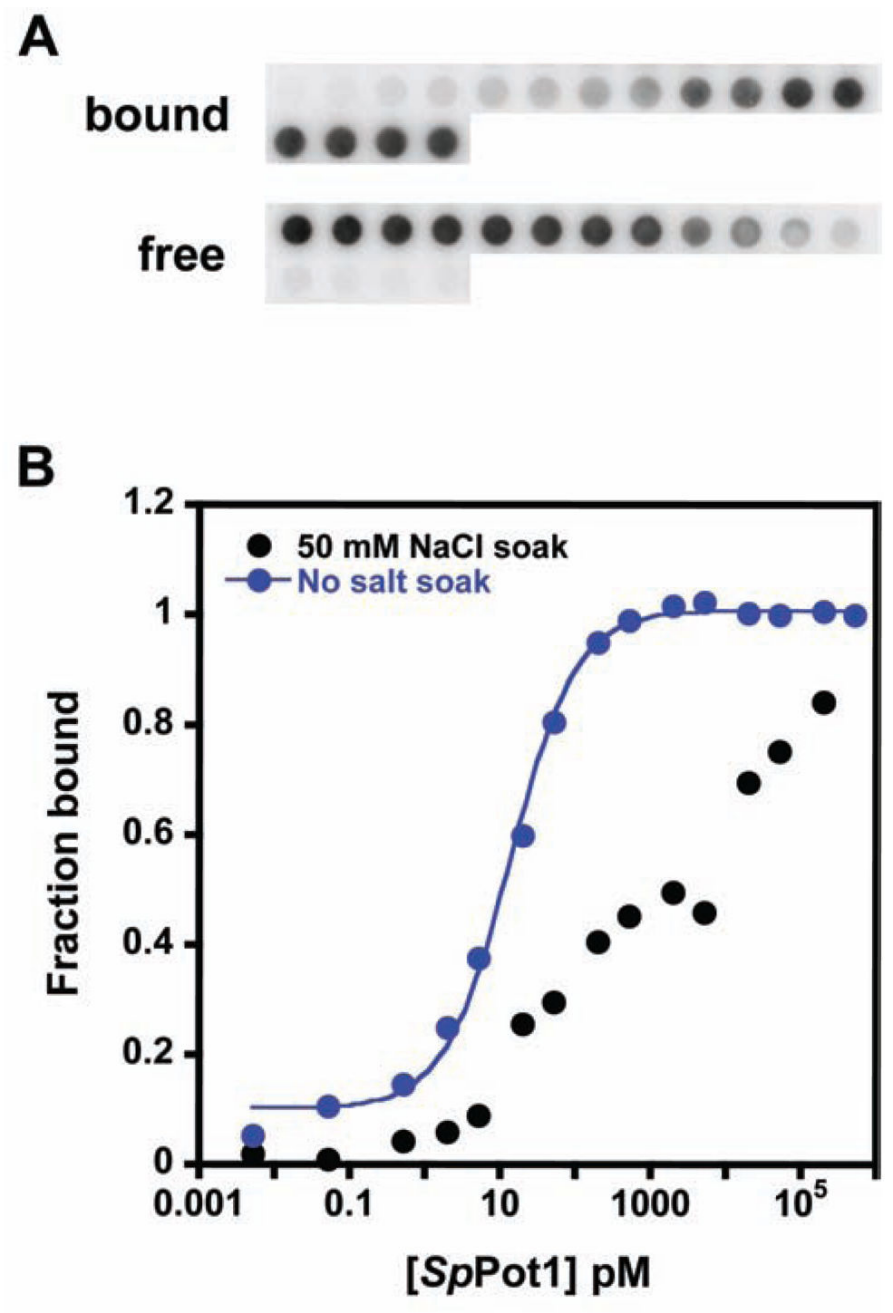
Example double-filter binding data and demonstration of the effects of equilibration of filters in salt-containing buffer. (A) Representative raw counts of nitrocellulose (bound) and Hybond XL (free) filters. *Sp*Pot1 was titrated at 5, 50, and 500 fM, 2, 5, 20, 50, 200, and 500 pM, 2, 5, 20, 50, 200, and 500 nM, with the first reaction containing no protein. (B) Identical binding reactions for *Sp*Pot1 and 1.5 pM 12mer were loaded onto filters pre-equilibrated in buffer (50 mM Tris, pH 8.0, 5 mM DTT) with 50 mM NaCl (black) and without (blue). Only data from filters equilibrated in salt-free buffer could be fit to a two-state binding model (Equation 1). Data are from a single representative experiment.

The double-filter binding method determines the fraction of nucleic acid bound to protein by comparing protein-bound nucleic acid captured by a nitrocellulose filter, to unbound nucleic acid, which passes through the nitrocellulose and is captured by a positively charged membrane. We essentially follow the original protocol outlined by Wong and Lohman,^7^ but with some critical modifications. The 96-well dot blot apparatus consists of a collection reservoir with an outlet to connect to an external vacuum pump, a filter plate with drainage holes, and a top plate with O-rings. Sandwiched between the top and filter plates are a nitrocellulose filter for capturing protein/nucleic acid complexes, a charged nylon filter for retention of free nucleic acid (a change from the original protocol, which employed a DEAE membrane), and a piece of 3 mm chromatography filter paper to cushion the filters against the filter plate, which obviates the need to replace the filter plate with a second top plate.^7^ In a significant revision of the original protocol, the nitrocellulose membrane is not pre-treated with any base soak or acid wash. Both membranes and the filter paper are equilibrated together in a salt-free version of the reaction binding buffer containing only the buffering and reducing agents for a minimum of 1 hr before being assembled with the apparatus. The filters are washed before and after sample loading with the equilibration buffer. Samples are loaded into the wells using a multi-channel pipettor, and, following application of vacuum to remove all liquid, the nitrocellulose and HyBond filters are dried with a hot-air gun and exposed to a phosphorimaging screen for quantification. The fraction of nucleic acid bound is calculated by dividing the counts from the nitrocellulose filter by the sum of the nitro-cellulose counts and the free nucleic acid counts from the HyBond filter (Figure 2A).

There are several variables specific to double-filter binding that require optimization. The faster off-rates that generally accompany lower-affinity interactions require careful verification that the volume and number of wash steps following the application of samples do not alter the *K*_D,app_. However, for high-affinity interactions (low picomolar *K*_D,app_), complexes typically do not dissociate on the time-scale of the experiment, and so the washing parameters are rarely a concern. In contrast, our studies have identified the composition of the filter equilibration buffer (which is typically used as the wash buffer as well) as a crucial factor that has a major impact on data quality. As mentioned above, the nitrocellulose and HyBond filters are soaked in a salt-free version of the binding buffer prior to assembly of the apparatus. We used the *Sp*Pot1/12mer interaction (Table 1) as a test and prepared two identical sets of binding reactions in 50 mM Tris, pH 8.0, 5 mM DTT (Tris/DTT), 50 mM NaCl, and 1 mg/mL BSA. We then directly compared the results of using sets of filters soaked in either Tris/DTT or Tris/DTT plus 50 mM NaCl. We observed a drastic difference in the measured binding activity (Figure 2B). Data from binding assayed with filters soaked in salt-free buffer is able to be fit to the sigmoidal curve to yield a *K*_D,app_. However, the data from the salt-soaked filters does not fit the binding isotherm, as saturation is not reached even at protein concentrations 10^5^-fold above the established *K*_D,app._^8^ Although we do not know the mechanism behind this phenomenon, the binding ability of both filters is affected. Therefore, when designing double-filter binding experiments, the equilibration buffer components should be evaluated for effects on *K*_D,app_ and binding saturation.

### General experimental design

#### Nucleic acid target considerations

**Figure 3:**
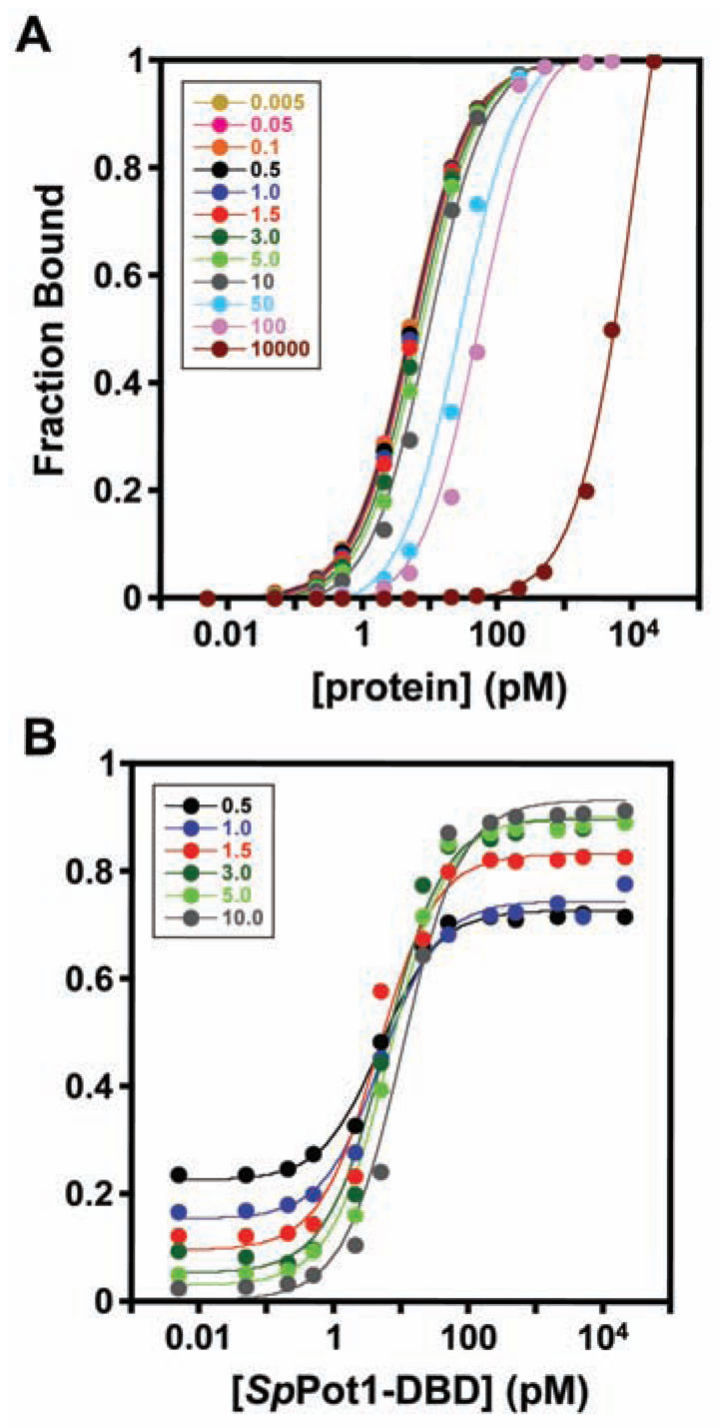
Nucleic acid target concentration must be set below the *K*_D,app_. (A) Simulated data set showing fits for calculating *K*_D,ap_ terms at various concentrations of target, both below and above a set KD of 4.9 pM (mean non-activity corrected KD,app value for the *Sp*Pot1-DBD/15mer interaction, see Table 1). Simulated data sets were calculated as described in Design and Methods. The curves shown are least-squares fits of Equation 1, and the fitted *K*_D,ap_ term values are reported in Table 2. (B) Non-background corrected fits of data obtained from binding reactions of *Sp*Pot1-DBD and 15mer at various ssDNA concentrations, as evaluated by EMSA. *Sp*Pot1-DBD concentrations ranged from 5 fM to 20 nM. Fraction of nucleic acid bound is plotted as a function of protein concentration and fit using a two-state binding model (Equation 1). Fitted *K*_D,app_ values are reported in Table 2.

It is critical to maximize the signal of the target in order to detect the femtomolar to picomolar concentrations of nucleic acid required for measuring *K*_D,app_s in the picomolar range. The most sensitive method for detection of protein/nucleic acid interactions is radiolabeling of nucleic acid, typically with ATP[γ-^32^P]. However, maximizing the signal must be balanced with the requirement that [D]_T_ ≪ *K*_D_, so that the assumption [P]_Free_ ≈ [P]_T_ will be valid. Therefore, the appropriate concentration of oligonucleotide must be carefully assessed. Using a nucleic acid concentration that is too high is one of the most commonly made mistakes in EMSA and filter binding experiments. If [D]_T_ > *K*_D_, [P]_free_ does not approximate [P]_T_. Therefore, because we are not directly measuring [P]_free_, the binding constant cannot be determined using Equation 1. The effect of a variety of nucleic acid target concentrations on the *fitted value* of the *K*_D,app_ term can be seen by simulating binding data for a range of target concentrations above and below the actual *K*_D,app_. As described in Design and Methods, we created 12 sets of simulated data for *Sp*Pot1-DBD binding to 15mer (actual *K*_D,app_ = 4.9 ± 0.8 pM, Table 1) using a range of oligonucleotide concentrations between 5 fM and 10 nM. Equation 1 was rearranged to define the fraction of nucleic acid bound solely in terms of the *K*_D,app_, the total ssDNA concentration, and the total protein concentration to yield Eq. 2:

(2) $$\frac{\left[\text{DP}\right]}{{\left[D\right]}_{T}}=O+S\left(\frac{({\left[D\right]}_{T}+N{\left[P\right]}_{T})-\sqrt{{\left({\left[D\right]}_{T}+N{\left[P\right]}_{T}\right)}^{2}-4N{\left[D\right]}_{T}{\left[P\right]}_{T}}}{2{\left[D\right]}_{T}}\right)$$

where [D]_T_ is the target nucleic acid concentration, [P]_T_ is the titrant protein concentration, [DP]/[D]_T_ is the fraction of nucleic acid bound, and *K*_D,app_ is the apparent dissociation constant. While the true *K*_D,app_ of the system is independent of protein and target nucleic acid concentrations, the fitted *K*_D,app_ term increases as a function of the target concentration (Figure 3A and Table 2). As [D]_T_ approaches 10-fold below the actual *K*_D,app_, the fitted value for the *K*_D,app_ term differs from the actual by ~3%, and when [D]_T_ ≈ *K*_D,app_, the fitted *K*_D,app_ is ~50% greater than the actual.

We also experimentally determined the fitted *K*_D,app_ for *Sp*Pot1-DBD binding to 15mer using a range of oligonucleotide concentrations between 0.5 and 10 pM. Because labeled oligonucleotides are recovered to varying degrees following spin cleaning, the absolute concentration of ssDNA is not known. We conservatively assume 80% recovery of 11–16 nucleotide-long ssDNA following spin-column elution; in general, longer ssDNAs are recovered to a greater extent from the column. Using this assumption, we have observed that the signal-to-noise ratio becomes too small for reliable quantitation below [D]_T_ ≈ 0.5 pM when using the most radioactive ATP[γ-^32^P] that is commercially available; see Design and Methods section. The fits of the raw data illustrate the increase in background noise that accompanies a decrease in ssDNA concentration as elevated baselines (Figure 3B). Experimental ssDNA concentrations between 0.5 and 1.5 pM yield fitted *K*_D,app_ values within the experimental error of the assays (for reference, we routinely obtain standard errors that are ~10% of the fitted *K*_D,app_; Figure 3B and Table 2). In contrast, at target ssDNA concentrations above 3.0 pM, the fitted *K*_D,app_ term increases with the concentration of target. Using both the *Sp*Pot1 and Cdc13 systems (data not shown), we have determined that ssDNA concentrations at or below 33% of the actual *K*_D,app_ allow replicable, consistent measurement of *K*_D,app_s. However, when working with a new system, one of the first experiments requires an assumption about the approximate *K*_D_ and survey several concentrations of target around that assumption. The fitted values of *K*_D,app_ will inform on the accuracy of that initial assumption. Subsequent experiments can be designed based on these results, although multiple iterations of this process may be needed to determine the optimal concentration of nucleic acid for a particular system.

### Protein considerations

Highly purified protein is a prerequisite for measuring accurate *K*_D,app_s. Contaminants in crude extracts and soluble aggregates of the protein of interest can directly affect the *K*_D,app_ and significantly alter protein activity. Thus, the titrant protein should be purified from the cell extract and gel filtration applied as a final purification step to remove aggregated protein. In all elements of preparing the protein for the binding reactions, both accuracy and precision are critical. When working with the very low concentrations required for tight-binding systems, even small errors and miniscule changes in protein molarity will drastically affect the system being studied. For example, a reduction in concentration by the protein adhering to the plastic of pipette tips and tubes can alter the measured *K*_D,app_. Using tips and tubes made of low-binding polypropylene helps address this issue. The concentration of the protein stock solution should be determined using at least two replicates, ideally by intrinsic UV absorption. An accurate experimental molar extinction coefficient can be obtained via denaturation.^29^ The use of Bradford assays is less accurate, as the inherent error in the assay significantly affects apparent concentrations. Additionally, an appropriate range of protein concentrations, from well below the *K*_D,app_ to beyond the saturation point of binding, is needed in order to determine accurate *K*_D,app_ values. Incomplete titrations, including titrations that have an insufficient number of data points in the transition region, may compromise the accuracy of the fitted value of *K*_D,app.20_ To ensure replicability, a minimum of three independent and precise sets of protein dilutions should be used for the determination of an average *K*_D,app_.

### Sample binding buffer optimization

For very tight binding regimes, the protein/nucleic acid interaction can be particularly sensitive to perturbation by a variety of factors, including buffer conditions, temperature, and time. Because tight binding interactions often involve fast *k*_on_ and slow *k*_off_ rates, it can take several hours to fully reach equilibrium. However, long incubation times must be balanced with considerations for protein stability, as binding activity and protein stability are closely related. Therefore, when designing a sample buffer for the binding reaction, one needs to identify specific conditions that generally promote stability of the protein. Parameters to consider include salt concentration, the buffering reagent, glycerol, and stabilizing additives like EDTA^2−^ and BSA. As discussed below, the sample buffer can have a profound effect on the measured binding affinity and should be optimized as part of experimental design. Two elements must be evaluated during the optimization process: protein activity and band quality, of which the latter can only be assessed via EMSA.

Significantly, only low salt buffer concentrations are suitable for EMSA to allow even electrophoresis through the gel. Because the electrophoresis running buffer must be devoid of salt, the ionic environment experienced by the protein/nucleic acid sample changes while being separated through the gel. In the sample buffer, total salt concentrations below 150 mN have been effectively used in our laboratory (K.A.L. and D.S.W unpublished results).^8,13,15,17^ *K*_D,app_ values for protein/DNA interactions are frequently dependent upon salt concentration.^30,31^ Therefore, for direct comparison of oligonucleotide binding constants and protein constructs, it is essential that the salt concentration in all experiments is identical.

Evaluating extremely tight protein/nucleic acid interactions can sometimes be made more experimentally tractable by increasing the ionic strength of the binding buffer, which will increase the *K*_D,app_. This allows the use of higher concentrations of radiolabelled nucleic acid, making detection much easier. In order to do this while retaining the ability to extrapolate to lower ionic strength environments (such as those found in the cell), however, one needs to empirically confirm the relationship between the *K*_D,app_ and salt concentration.^31–33^ Despite the potential advantages of increasing salt concentration, it is not a universal solution to studying tight-binding systems. In some cases, changes in salt can result in changes in oligonucleotide length preference;^34–36^ in other situations, such as with *Sp*Pot1, salt concentration affects the binding mode utilized for interaction with the ssDNA target.^8,13^

**Figure 4:**
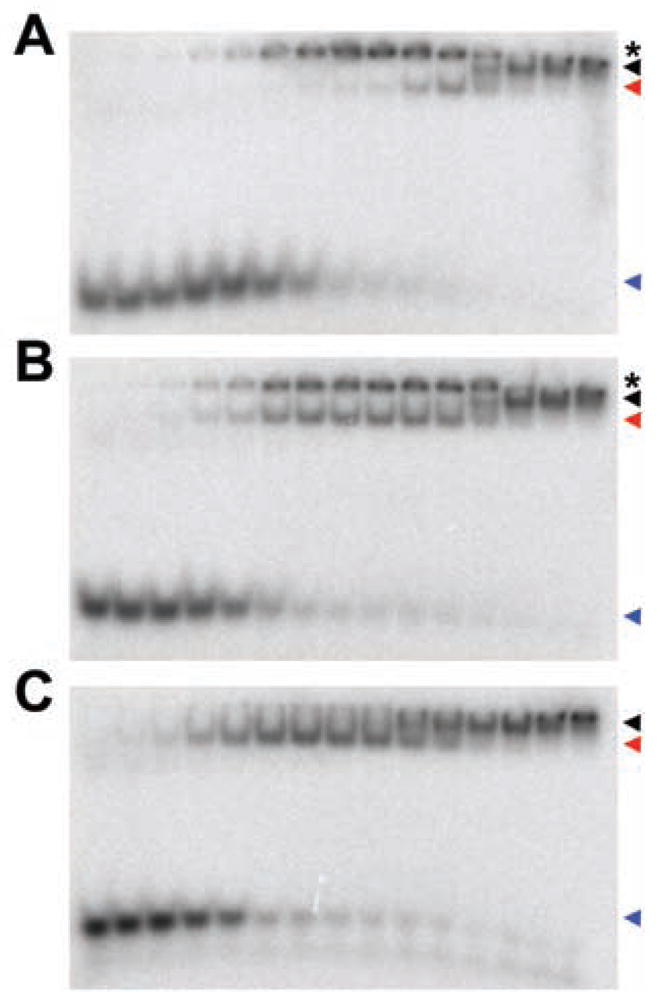
Effects of different buffering agents in the sample buffer. EMSAs were performed using *S. cerevisiae* Cdc13 at the same concentrations as in Figure 1A and 1 pM Tel11 target ssDNA in reaction buffers made with 50 mM of either (A) HEPES, (B) tricine, or (C) potassium phosphate at pH 7.8. All gels were run at 200V for 35 minutes at 4°C. Both the HEPES and tricine buffers produced a prominent well-shift (*) at middle protein concentrations, which indicate aggregation of the protein/ ssDNA complex. In contrast, the potassium phosphate buffer produced data indistinguishable from those obtained using a Tris-HCl reaction buffer (Figure 1A, bottom right panel). Red and black arrows indicate the monomer and dimer species, respectively, and blue arrows indicate free ssDNA.

The buffering agent used in the binding reaction is also a critical variable that can be effectively optimized for the protein/nucleic acid system being studied. Proteins can be prone to condition-dependent aggregation, as observed by a portion of the protein remaining in the wells (Figure 4, asterisks). For example, HEPES and tricine are incompatible buffering agents with the Cdc13/Tel11 system (Figure 4A and 4B). In contrast, the protein appears far more stable and soluble in sample conditions containing either potassium phosphate (Figure 4C) or Tris-HCl (Figure 1A, bottom right panel) buffered to the same pH. In addition to the aggregated well-shifted species, the measured *K*_D,app_ is five-fold weaker in the HEPES reaction than in the other buffers, demonstrating that the choice of buffer also has a direct effect on the binding activity of the protein. As protein systems are idiosyncratic, each needs to be assessed for buffer compatibility.

We also investigated the effect of buffer additives on protein activity. While these components can be optimized for any binding assay, here we use filter binding to demonstrate how additives can alter the measured *K*_D,app_ of *Sp*Pot1 and 12mer. We have found that the addition of BSA substantially improves the activity of various telomere end-protection proteins (S.E.A, K.A.L., D.S.W. unpublished data).^13^ *In vitro*, many of these proteins tend toward aggregation in the absence of their ssDNA ligands. BSA likely functions to prevent aggregation as well as to saturate potential protein binding sites on plastic surfaces, and in doing so increases the fraction of active protein in solution. In order to characterize the effect of BSA on protein activity, we performed binding experiments with *Sp*Pot1 and 12mer in a set of binding buffers either 0.1 μg/mL or 1 mg/mL BSA. We observed a 7-fold decrease in the measured *K*_D,app_ with the inclusion of 1 μg/mL BSA in the binding buffer (Table 3). These data support our hypothesis that BSA helps maintain a higher proportion of active protein.

Historically, protein/nucleic acid binding studies have often been conducted in the presence of non-specific nucleic acid, such as poly(dI/dC), salmon sperm DNA, or tRNA.^18,37^ The inclusion of non-specific nucleic acids is advantageous when assays are performed with crude extracts or impure protein preparations, in order to prevent the specific target from being bound by non-specific nucleic acid binding proteins that may be present. However, non-specific nucleic acids are not necessary for studies involving highly purified proteins, and can even be detrimental to the interaction. We tested the effect of the addition of 0.1 mg/mL tRNA on binding activity in our *Sp*Pot1/12mer assay. We found that, while tRNA had a moderate effect on binding activity in the presence of 1 mg/mL BSA, its addition exacerbated the effects of low BSA concentration, increasing the *K*_D,app_ by 10-fold (Table 3). In this system it appears that tRNA negatively impacts protein activity, thus the need to add of non-specific nucleic acids to binding assays with purified nucleic-acid binding proteins should be evaluated.

The above *Sp*Pot1 experiments were all conducted in the absence of salt. While some proteins remain fully active in salt-free buffer, the majority of proteins do not. Low concentrations of salt are often required as a co-solvent for proteins in aqueous solution, balancing the surface charges of protein residues and creating a stable hydration shell.^38^ With the addition of 50 mM NaCl to the 1 mg/mL BSA binding buffer, we observed a 7-fold decrease in *K*_D,app_ (Table 3). Taking all of the condition variables into consideration, these additives can produce *K*_D,app_ values ranging over three logs (Table 3). These results establish the importance of assessing the effects of sample buffer additives on binding activity before proceeding with quantitative binding assays. Furthermore, this example clearly highlights the issues associated with direct comparison of absolute *K*_D,app_ values determined using different experimental conditions.

### Data processing

#### Quantitation

**Figure 5:**
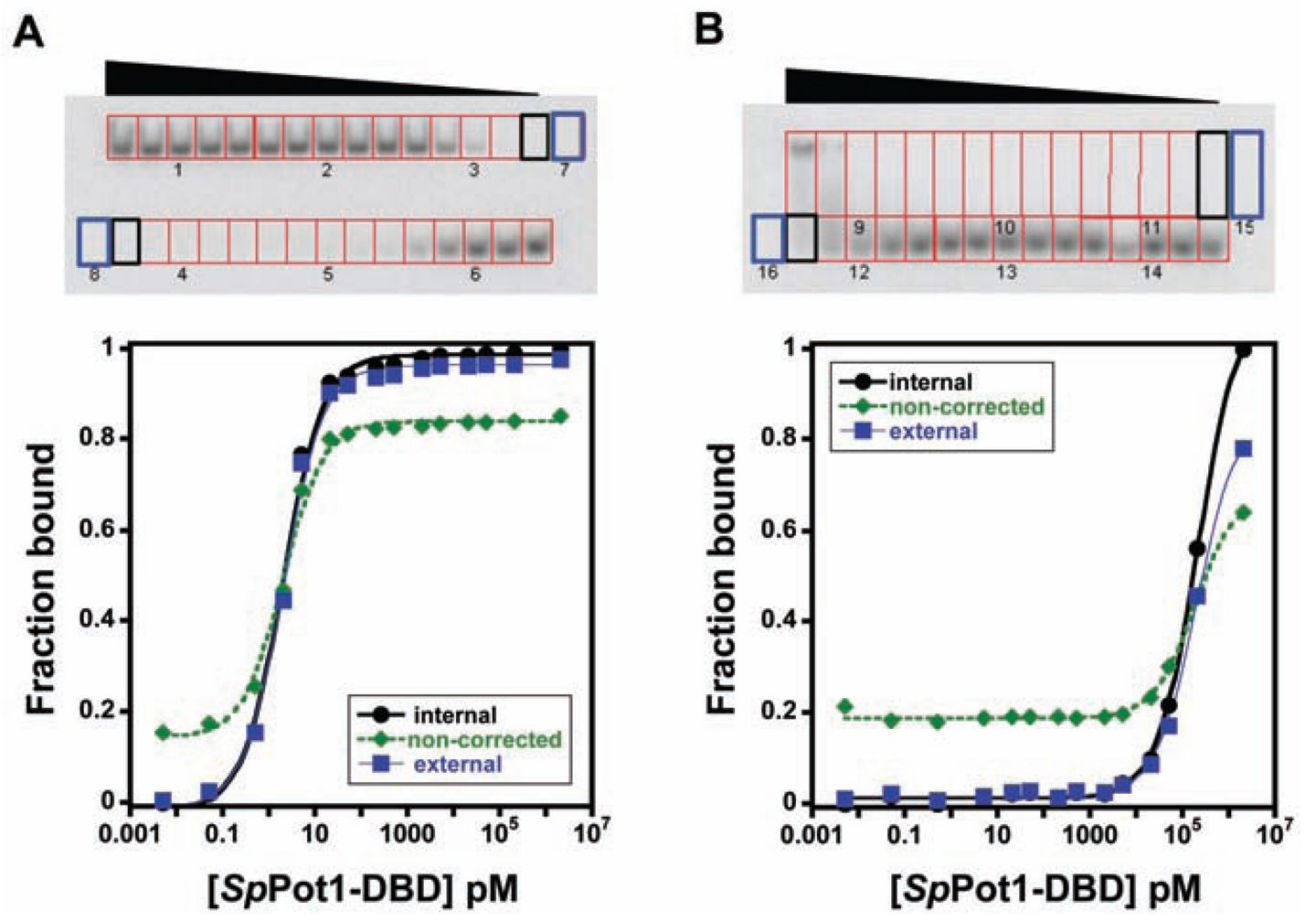
Data quantitation and background correction. (A) Gel and quantitation of a tight binding interaction (*Sp*Pot1-DBD/15mer). (B) Gel and quantitation of a weak binding interaction (*Sp*Pot-DBD/d(GGTTA)). For both binding reactions, *Sp*Pot1-DBD was titrated using 5, 50, and 500 fM, 2, 5, 20, 50, 200, and 500 pM, 2, 5, 20, 50, and 200 nM, and 2 μM protein with oligonucleotide held at 1.5 pM. For quantitation, each band is boxed individually. Note that for the weak binding interaction, the “bound” boxes are larger, in order to measure all counts that exhibited lower mobility. Additionally, three background subtraction methods are demonstrated: internal, external, and none. For internal correction, the counts from the blue boxes were subtracted from the corresponding sets (blue curve). For external correction, the counts in the black boxes were subtracted from the corresponding sets (black curve). Finally, no background correction was performed at all (green curve). *K*_D,app_ values as determined by fitting Equation 1 are shown.

As discussed above, tight binding interactions require very small amounts of labeled nucleic acid. The low level of radiolabel requires that the dried gels and filters be exposed to phosphorimaging screens for extended periods of time (2–3 days). As a result, there is always a low level of background signal, which is not mitigated by further extending the exposure time. The combination of relatively weak radiolabel signals and significant measurable background presents signal-to-noise issues that need to be considered for such tight-binding systems. First, areas are defined around the unbound and bound bands for each concentration of titrant. To reduce background signal, the areas should be defined to incorporate all positive signal while minimizing areas that do not contain nucleic acid counts. For interactions that have slow off-rates and which do not dissociate during the run time of the gel, the areas for the bound and unbound bands can be of equal size, which allow the counts to be directly compared. *Sp*Pot1-DBD bound to 15mer demonstrates a clear distinction between bound and unbound species (Figure 5A). In contrast, interactions with faster off-rates may dissociate during the gel run. In order to capture all of the bound species that existed at the beginning of the experiment, the quantitation area for the bound species needs to encompass a larger portion of the lane, extending from the slowest-migrating species at the top to just above the unbound target band at the bottom of the gel. In this way, bound species that dissociate during the running of the gel and create a smear will be accounted for as initially bound species. For example, a five-nucleotide ssDNA oligonucleotide binds to *Sp*Pot1-DBD with a relatively fast off-rate, causing a smear in the gel (Figure 5B). To facilitate background correction, as discussed below, each quantitation area should be identical (*i.e*., use the same size box for all bound species in one gel).

Additionally, the signal-to-noise ratio decreases with decreasing oligonucleotide concentration (Figure 3B). Therefore, quantitation of the data requires background correction, which can be conducted using one of two methods: external or internal correction. External correction measures the background counts in an equally sized area next to the gel-or filter-exposed section and subtracts that value from all data points (Figure 5, blue boxes). Internal correction uses the counts from either end of the titration as the background value, which is then subtracted from all other points (Figure 5, black boxes). Figure 5 shows these two quantitation methods applied to either a high-affinity (10^–12^ M) or a lower-affinity (10^–8^ M) interaction. For the high-affinity interaction, there is no difference in the measured *K*_D,app_s using either correction method, nor do those values differ from the *K*_D,app_ measured from non-corrected raw data (Figure 5A). However, the choice of quantitation and background-correction method can impact the resulting *K*_D,app_s for lower-affinity interactions. Since the target is never fully saturated (unbound target remains at the highest titrant concentration), the internal correction method is not appropriate (Figure 5B). The external correction method accounts for the true background counts, and when plotted and fitted, the curve correctly reflects that saturation was not reached. Because the external correction method works well for all titrant/target interactions, it is a reliable *default* for the range of *K*_D,app_s measured by EMSA and double-filter binding.

#### Activity correction

**Figure 6:**
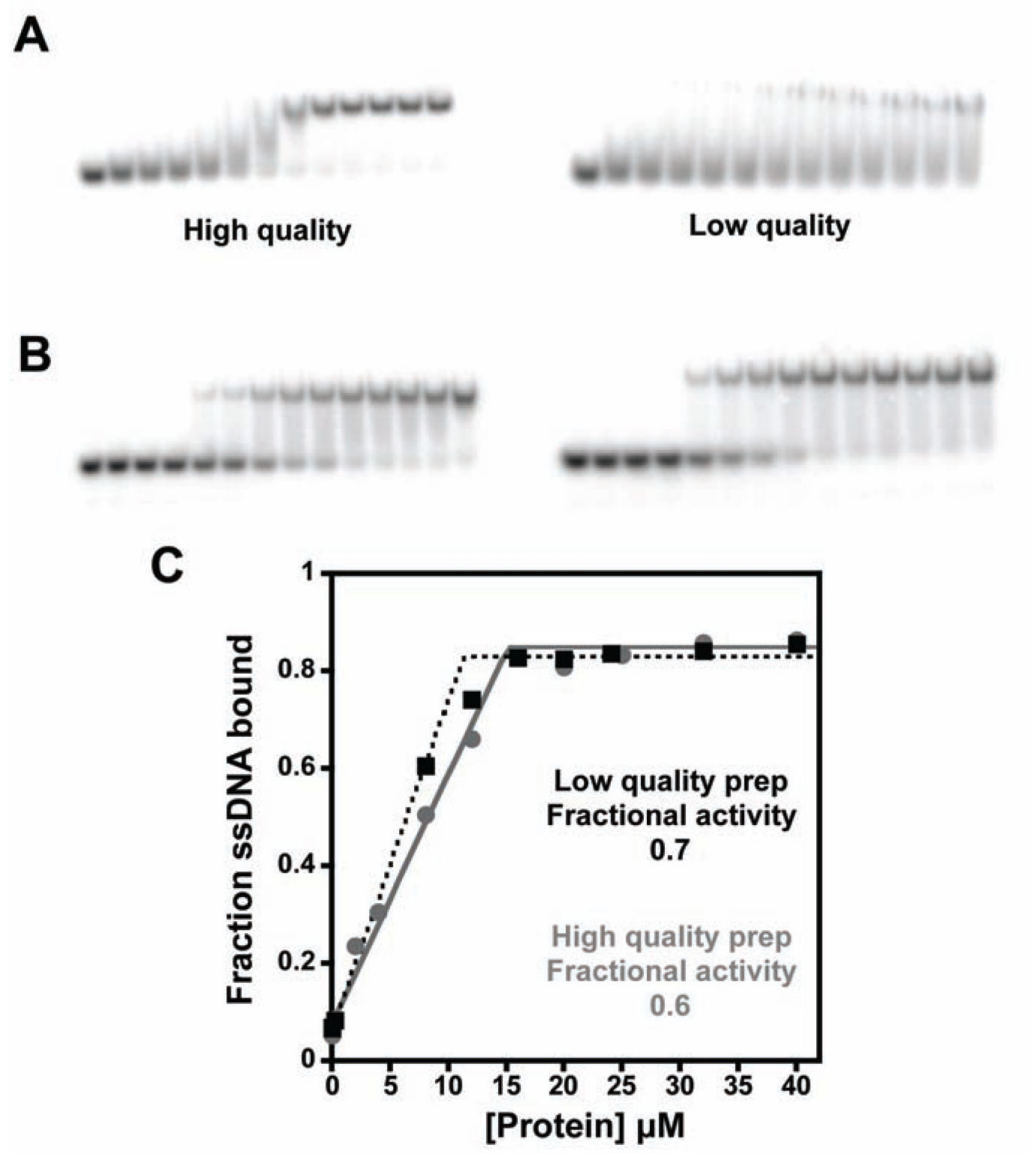
The activity correction assay does not necessarily report on the activity of a protein in a binding assay. (A) Representative EMSA binding gels showing quality of data obtained using the same concentrations (2.5, 25, 62.5, 125, 250, and 625 nM, 1.25, 2.5, 6.25, 12.5, 25, 37.5, and 62.5 μM) of two different preparations of Pot1pC binding to 50 pM ssDNA. (B) Representative EMSA activity gels for the same protein preparations as in (A), using ssDNA at 4 μM (+ 50 pM ^32^P-labeled) and Pot1pC concentrations of 200 pM, 2, 20, and 200 nM, 2, 4, 8, 12, 16, 20, 25, 32, and 40 μM. (C) Quantitation and fitting of data from (B) demonstrates that poorly behaved protein can be active in the presence of high target concentrations required for the activity assay. Activity assay data were fit using Equation 3.

Measurement of the active concentration of titrant protein allows reproducible *K*_D,app_ values to be measured from multiple preparations of protein. Activity assays are designed to take into account inaccurate determination of total protein concentration due to erroneous molar extinction coefficients and the presence of inactive protein.^20^ Additionally, determining the fractional activity of a protein sample, or the concentration of protein in solution that is functional, is important for assaying the effects of protein mutations on binding activity. To measure active protein concentration, the nucleic acid is held constant at a concentration well above the *K*_D,app_ of the interaction and protein is titrated at concentrations well below and above the nucleic acid concentration. Note that the majority of the nucleic acid target for this experiment is unlabelled. Because the labeled oligonucleotide is not fully recovered following spin cleaning, the absolute concentration cannot be easily determined. Thus, unlabeled nucleic acid is used, with a negligible amount (≤0.05%) of ^32^P-labeled target included for quantitation purposes. Importantly, the stoichiometry of the protein/nucleic acid system must be known, as this will impact the saturation point. As mentioned above, the systems discussed here have 1:1 stoichiometry. For the *Sp*Pot1 system, we typically use 100 nM ssDNA (for *Sp*Pot1/15mer this is ~25,000-fold above the measured *K*_D,app_), and protein is titrated over a range of 1 pM to 1 mM. Ideally, with 100% active protein in this 1:1 binding system, saturation would be reached at 100 nM *Sp*Pot1. However, in practice, the fractional activity of a protein will rarely be 1. The data are plotted as a linear function of fraction of nucleic acid bound versus protein concentration. In our laboratory, we then perform a least-squares fit using the equation:

(3) $$\frac{\left[\text{DP}\right]}{{\left[D\right]}_{T}}=O+S\left(\frac{(K_{D,\text{app}}+{\left[D\right]}_{T}+{\left[P\right]}_{T})-\sqrt{{\left(K_{D,\text{app}}+{\left[D\right]}_{T}+{\left[P\right]}_{T}\right)}^{2}-4{\left[D\right]}_{T}{\left[P\right]}_{T}}}{2{[D]}_{T}}\right)$$

where [DP] is the concentration of complex, [D]_T_ is the total concentration of nucleic acid used, S is a saturation offset, N is the number of sites occupied, [P]_T_ is the total protein concentration, and O is the background offset. We and others have used this method to determine the active concentration of a variety of wild type and mutant proteins.^8,13–15,17,39^ The fractional activity is the ratio of target:protein concentration at which the fraction of nucleic acid bound reaches saturation, defined as the protein concentration at which the fitted curve contains a discontinuity, which is clearly seen when the data are plotted linearly. The experiment should be performed with multiple replicates, using the average of all replicates as the fractional activity value. We typically obtain fractional activity between 0.6 and 1 (Figure 6C). For data quantification, the protein concentrations used in the *K*_D,app_ experiments are multiplied by the fractional activity value to determine the concentration of active protein present in the interactions with the target.

The activity assay accounts for *irreversibly* inactive protein. However, when proteins are *reversibly* inactive, the activity assay alone does not provide a measure of active protein concentration. In the binding experiment, the majority of the protein is in the free state at all times, whereas in the activity correction experiment the protein is fully bound. Consider a protein for which the bound form is significantly more stable than the free form. In this case, the binding of target to the protein may convert some inactive to active protein and results in an incorrect assessment of the fractional activity of the protein. In some cases, the gel of an activity assay can visually report on whether the inactive protein is restored upon target binding. This phenomenon can be observed by comparing a high-quality preparation of protein to a poor-quality sample. For this example, we used a DNA-binding subdomain of *Sp*Pot1, Pot1pC, which displays lower affinity for cognate ssDNA (10^−8^ M) than *Sp*Pot1.^8,12^ The differences in protein stability and activity are readily distinguished in EMSAs designed to determine the *K*_D,app_, in which [D] ≪ [P] and thus [P]_free_ ≫ [PD], as the low-quality prep exhibits a weak shift to the bound form and smeary lanes (Figure 6A). However, the difference in protein quality is not discernable in the activity assays, which both show sharp bands and good fits to the activity-correction equation (Figure 6B and C). Because the activity assay saturates the protein with target nucleic acid at even the lowest protein concentrations, it can mask the presence of unstable/less-active protein and yields a higher apparent fractional activity than exists for the free form that is present in the *K*_D,app_ experiments. This phenomenon is also observed with full-length *Sp*Pot1, for which we measured similar fractional activities, but *K*_D,app_s that differ by orders of magnitude between different buffers (Table 3 and data not shown). Therefore, this assay should be only one of several assays used to evaluate the overall quality of a protein preparation.

## Discussion

EMSAs and double-filter binding are powerful strategies that are widely employed in probing nucleic acid interactions. These techniques have advantages and disadvantages to be considered during assay development. EMSAs visually report on several binding features beyond measurement of the binding constant. An EMSA will qualitatively report on the off-rate of a protein/nucleic acid interaction in the form of a visible smear between the unbound and fully bound bands as the protein and oligonucleotide dissociate (Figure 5). In contrast, a fast off-rate can artificially inflate the *K*_D,app_ as measured by a double-filter binding assay, because any and all free nucleic acid will pass through the nitro-cellulose to be trapped by the HyBond filter, both when the sample is initially applied and during subsequent wash steps. Additionally, because EMSA separates species according to electrophoretic mobility, it may be possible to distinguish different oligomeric states of bound species and/or conformations that are not distinguishable by double-filter binding.^6,40^ In our laboratory, we have used EMSAs to identify different binding states for *Sp*Pot1 proteins and monomeric versus dimeric Cdc13 binding (Figure 4).^8^ Finally, the visual feedback provided by EMSAs enables a qualitative assessment of protein preparation quality, including aggregated proteins retained in the well (Figure 4) and reversible inactivity (Figure 6).

Double-filter binding assays offer the advantage of being relatively high throughput compared to other methods, including EMSA.^7^ As the rapidity of both the method and quantitation are key to large-scale binding studies, this assay can be ideal for such an application. The double-filter binding assay is typically conducted in a 96-well dot blot format, which greatly increases the number of samples that can be analyzed in each experiment. Additionally, the application of samples and separation of unbound and bound species are quite fast, especially when samples are loaded into the apparatus using a multi-channel pipettor. Additionally, while both filters and gels must both be dried prior to exposure to phosphorimaging screens in order to prevent diffusion of protein and nucleic acid, the drying time for filters is only a fraction of the time required for gel drying. This short time allows binding reactions that were incubated on ice to be loaded at room temperature. Finally, because double-filter binding does not involve any electric current, both positively and negatively charged protein systems can easily be assayed, and the technique is highly amenable to assessing binding activity in high-salt buffers. Another advantage provided by double-filter binding that is especially relevant to high-affinity protein/nucleic acid interactions is that much larger sample volumes can be analyzed, which can help offset the low signal of a small concentration of radiolabeled nucleic acid. Samples of over 150 μL can be loaded, allowing for the reliable measurement of sub-picomolar affinities. In contrast, mini-gel EMSAs allow for the analysis of at most 30 mL of sample, with a lower limit of measuring *K*_D,app_s of 2–4 pM.

Our work with telomere-end systems has enabled the identification of several conditions and parameters to be considered when designing EMSA and double-filter binding experiments for unusually tight protein/nucleic acid interactions. We present a number of practical experimental considerations that have allowed us to reproducibly measure accurate *K*_D,app_s in the low picomolar range by both EMSA and double-filter binding. This has been achieved by using the freshest lot of ATP[γ-^32^P] that is commercially available and thorough optimization of the binding conditions. We suggest that EMSA is generally a more appropriate assay for the initial characterization of protein/nucleic acid interactions, and, if a single bound species is observed, can be followed by evaluation with double-filter binding as a higher throughput technique, as long as the *K*_D,app_s determined by both methods are in agreement. We also find that the measurement of *K*_D,app_ values for high-affinity systems can be very sensitive to subtle alterations of binding conditions and technique. Therefore, *K*_D,app_ values acquired under different experimental conditions cannot be directly compared. However, with careful experimental design, binding constants in the picomolar regime can be confidently and reproducibly determined.

## Figures and Tables

**Table 1:** Protein/ssDNA binding systems discussed.

Protein construct (residue span)	ssDNA target	K_D,app_ (pM)*
*Sp*Pot1 (1–555)°	12mer d(GGTTAC)2	6.7±0.9#
	15mer d((GGTTAC)2GGT)	5.2±0.5#

*Sp*Pot1-DBD (1–389)	15mer d((GGTTAC)2GGT)	4.9±0.8#

Cdc13 (1–924)°	Tel11 d(GTGTGGGTGTG)	13.4±0.8

* Non-activity corrected values;

° full-length protein;

# Activity-corrected values were previously reported.^8^

**Table 2:** K_D,app_ values for a range of target concentrations.

[Target] (pM)	Simulated fitted *K*_D,app_ (pM)*	Experimental fitted *K*_D,app_ (pM)°
0.005	4.90	-
0.05	4.92	-
0.1	4.95	-
0.5	5.15	5.0
1.0	5.39	5.1
1.5	5.64	4.5
3.0	6.38	5.8
5.0	7.3	6.8
10	9.53	10.3
50	27.9	-
100	54.6	-
10,000	12,000	-

* Simulated using true *K*_D,app_=4.9 pM;

° true *K*_D,app_=4.9±0.8 from triplicate EMSA experiments (Table 1).

**Table 3:** *K*_D,app_ values for *Sp*Pot1 binding to 12mer in different sample buffer conditions.

Buffer formulation	*K*_D,app_ (pM)*
Base buffer°	700
+ 1.0 mg/mL BSA	100
+ 0.1 μg/mL tRNA	7000
+ 1.0 mg/mL BSA, 0.1 μg/mL tRNA	130
+ 1.0 mg/mL BSA, 50 mM NaCl	14

* Values are from a single experiment;

° Base buffer is 50 mM Tris, pH 8.0, 1 mM DTT, 0.1 μg/mL BSA.
